# The potential and limitations of induced pluripotent stem cells to achieve wound healing

**DOI:** 10.1186/s13287-019-1185-1

**Published:** 2019-03-12

**Authors:** Jolanta Gorecka, Valentyna Kostiuk, Arash Fereydooni, Luis Gonzalez, Jiesi Luo, Biraja Dash, Toshihiko Isaji, Shun Ono, Shirley Liu, Shin Rong Lee, Jianbiao Xu, Jia Liu, Ryosuke Taniguchi, Bogdan Yastula, Henry C. Hsia, Yibing Qyang, Alan Dardik

**Affiliations:** 1 Vascular Biology and Therapeutics Program and the Department of Surgery, Yale School of Medicine, Yale University, 10 Amistad Street, Room 437, PO Box 208089, New Haven, CT 06520-8089 USA; 2 Section of Cardiovascular Medicine, Department of Internal Medicine, Yale Cardiovascular Research Center, Yale School of Medicine, 300 George Street, Ste 773A, New Haven, CT 06511 USA; 3 Section of Plastic Surgery, Department of Surgery, Yale School of Medicine, Yale University, PO Box 208062, New Haven, CT 06520-8062 USA; 4 Yale Stem Cell Center, Yale University, New Haven, USA; 5 Vascular Biology and Therapeutics Program, Yale School of Medicine, New Haven, USA; 6 Department of Pathology, Yale University, New Haven, USA

**Keywords:** Induced pluripotent stem cell, Stem cell, Chronic wounds, Wound healing, Diabetic foot ulcer, Peripheral arterial disease, Angiogenesis, Diabetes, Teratoma

## Abstract

Wound healing is the physiologic response to a disruption in normal skin architecture and requires both spatial and temporal coordination of multiple cell types and cytokines. This complex process is prone to dysregulation secondary to local and systemic factors such as ischemia and diabetes that frequently lead to chronic wounds. Chronic wounds such as diabetic foot ulcers are epidemic with great cost to the healthcare system as they heal poorly and recur frequently, creating an urgent need for new and advanced therapies. Stem cell therapy is emerging as a potential treatment for chronic wounds, and adult-derived stem cells are currently employed in several commercially available products; however, stem cell therapy is limited by the need for invasive harvesting techniques, immunogenicity, and limited cell survival in vivo. Induced pluripotent stem cells (iPSC) are an exciting cell type with enhanced therapeutic and translational potential. iPSC are derived from adult cells by in vitro induction of pluripotency, obviating the ethical dilemmas surrounding the use of embryonic stem cells; they are harvested non-invasively and can be transplanted autologously, reducing immune rejection; and iPSC are the only cell type capable of being differentiated into all of the cell types in healthy skin. This review focuses on the use of iPSC in animal models of wound healing including limb ischemia, as well as their limitations and methods aimed at improving iPSC safety profile in an effort to hasten translation to human studies.

## Introduction

Wound healing is a complex physiological response to the disruption in the normal architecture of the protective skin barrier. It involves the spatial and temporal coordination of various cell types and cytokines and is divided into three distinct phases: inflammation, proliferation, and remodeling [1, 2]. This complex and delicate process is prone to dysregulation secondary to local and systemic factors that can lead to failure of healing and progression to chronicity.

Immediately post-wounding, hemostasis is achieved by platelet aggregation and initiation of the coagulation cascade. Chemotactic signals including platelet-derived growth factor (PDGF) and fibroblast growth factor (FGF) are released and attract macrophages, neutrophils, and fibroblasts to the wound bed. Neutrophils and macrophages clear the wound of any bacteria and debris and elaborate further cytokines including interleukin 1 (IL-1), vascular endothelial growth factor (VEGF), and tumor necrosis factor alpha (TNF-α), all of which promote cellular recruitment and proliferation. A provisional matrix composed of fibronectin and hyaluronic acid (HA) is synthesized and secreted, upon which epithelialization occurs with the aid of stem cells from hair follicles and adjacent epidermis. Eventually, fibroblasts convert fibronectin-based microfibrils into collagen-enriched fibers. During these events, angiogenesis results in re-vascularization of the wound [3–6].

Chronic cutaneous wounds, such as those seen in diabetic foot ulcers (DFU) and pressure ulcers, contribute significantly to patient morbidity and mortality. They affect over six million Americans annually and cost upwards of $25 billion [7]. Nearly every stage of wound healing becomes dysregulated in diabetic wounds, contributing to the poor healing of DFU. Diabetic individuals suffer from impaired growth factor production, decreased angiogenesis, depressed macrophage function and collagen accumulation, poor keratinocyte and fibroblast migration and proliferation, and impaired stem cell homing [1, 8–11].

The gold standard of chronic wound management involves careful diagnosis of etiology, control of infection, optimization of vascular inflow to reduce ischemia, debridement of nonviable tissue, and offloading of pressure. Despite optimal care, only 50% of DFU heal within 12–20 weeks and 50% recur within 18 months. As such, novel and improved methods of wound healing are urgently needed [12–16].

Stem cell therapy has emerged as an exciting potential therapy for wound healing. When transplanted into a wound, stem cells act in a direct and paracrine manner to promote cell recruitment, immunomodulation, extracellular matrix remodeling, and angiogenesis by secretion of cytokines and growth factors [17–19]. Adult-derived cells, such as mesenchymal stem cells (MSC), have shown potential in accelerating healing of chronic wounds, particularly in the diabetic population where populations of MSC are deficient [20–24]. MSC have shown efficacy in multiple clinical trials of DFU healing and are currently included in several commercially available topical products including Grafix and Stravix [25–27]. Embryonic stem cell (ESC)-derived MSC are superior to adult-derived MSC, as they retain their potency, show a high proliferative ability, and display a consistent phenotype [28]. However, use of these cells is limited by the ethical issues associated with the use of embryonic stem cells, need for invasive harvesting techniques, immunogenicity, and limited cell survival in vivo [29].

## Induced pluripotent stem cells in chronic wound healing

Induced pluripotent stem cells (iPSC) represent a groundbreaking innovation for adult-derived stem cells that carry enhanced therapeutic and translational potential. First developed in 2006, iPSC are pluripotent stem cells derived from adult somatic cells. They are reprogrammed into a pluripotent state in vitro by induced expression of four transcription factors including Oct4/Sox2/c-Myc/KLF4 or Oct4/Sox2/NANOG/LIN28 [30–32]. Although their initial derivation required retroviral transfection, recent progress in stem cell techniques allows for their generation with the use of non-integrative techniques, improving their safety profile (Table 1) [33–35].

**Table 1:** Current methods for generating induced pluripotent stem cells

	MMLV-derived retrovirus	Lentivirus	piggyBac	Adenovirus	Sendai virus	Plasmid	Episome	Minicircle	RNA delivery	Protein delivery
Non-integrative	−	−	−	+	+	+	+	+	+	+
DNA-free	−	−	−	−	−	−	−	−	+	+
Efficient	++	++	+	−	+	+	+	+	++	–
Safe	–	–	+	+	+	+	+	+	++	++

Current methods of induced pluripotent stem cell derivation, their advantages, and limitations. Non-integrative: + yes, − no. DNA free: + yes, − no. Efficient: ++ high, + medium, − low. Safe: ++ high, + medium, − low

Similar to embryonic stem cells, iPSC are pluripotent, have the potential for self-renewal, and can differentiate into any adult cell type. iPSC have certain advantages over other stem cell types in models of regenerative medicine and wound healing. Because they are derived from adult somatic cells, and not embryos, iPSC are not associated with the ethical dilemmas surrounding the use of embryonic stem cells. They are easily harvested from cutaneous sources such as skin fibroblasts, obviating the need for invasive harvesting procedures such as bone marrow or adipose tissue biopsies. iPSC are pluripotent and can therefore be differentiated into any adult cell type, enhancing their potential in models of various disease processes. Since iPSC can be derived in principle from any adult tissue including skin, the potential pool of source cells is many orders of magnitude greater than other stem cell types. Lastly, iPSC can be transplanted in an autologous fashion to avoid immunogenicity, enhancing their in vivo survival [36, 37].

iPSC are currently being evaluated in pre-clinical studies of three-dimensional (3D) organ printing, wound healing, and angiogenesis [38]. Although much remains to be learned about their safety and generation methods prior to use in humans, iPSC are already being investigated in clinical trials of disease modeling including cardiomyopathy, autism spectrum disorder, coronary artery disease, oncology, and cystic fibrosis [38].

Because they can differentiate into descendants of all three germ layers, iPSC-derived terminally differentiated cells have the potential to enhance each of the phases of diabetic wound healing through their paracrine and direct cellular effects (Table 2) [38]. During the inflammatory phase, iPSC-derived cells can secrete growth factors and cytokines, counteracting the suppressed cytokine secretion profile seen in diabetic patients [39, 40]. This results in recruitment of macrophages as well as proliferative cells including fibroblasts and keratinocytes, which are known to be deficient in chronic wounds [41–44]. Direct application of stem cells into the wound bed also mitigates the impaired homing potential of progenitor cells into diabetic wounds [45]. In the proliferative phase, potential iPSC-derived cells include endothelial cells, smooth muscle cells, fibroblasts, pericytes, keratinocytes, or MSC [46, 47], subsequently promoting angiogenesis [43] and increasing collagen deposition [42]. Because the remodeling phase is highly dependent on functional myofibroblasts, their recruitment during the proliferative phase is essential to the last stage of wound healing. Finally, unlike MSC, iPSC retain the ability to differentiate into keratinocytes [46].

**Table 2:**
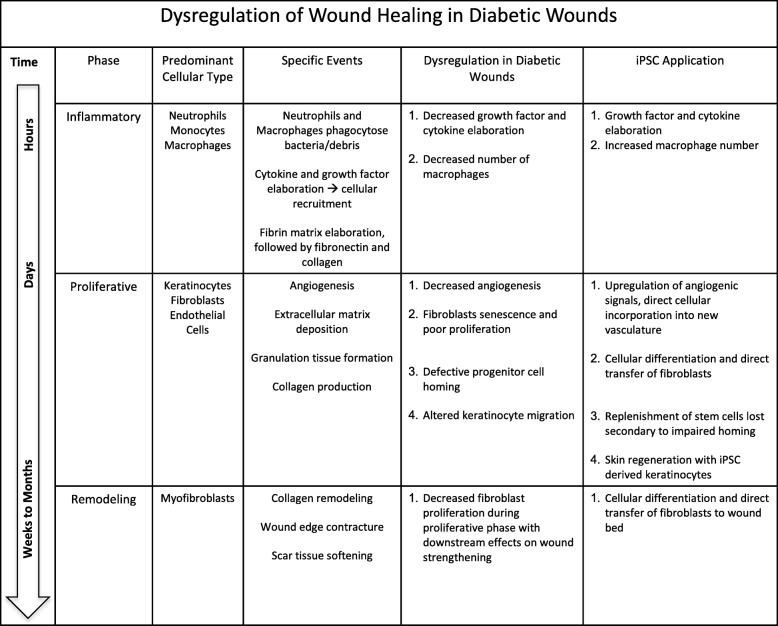
Dysregulation of wound healing in diabetic wounds

Dysregulation of normal wound healing process in diabetic wounds and effects of induced pluripotent stem cells on each phase. Adapted from Falanga [1]

The following sections review the use of iPSC in animal preclinical studies of non-ischemic and ischemic wound healing and the limitations that need to be overcome prior to clinical use. Major findings of studies relating to cutaneous wound healing in a murine model are outlined in Table 3.

**Table 3:** Induced pluripotent stem cells in wound healing

Author	Year	Cell type	Delivery method	Cell number	Animal model	Major findings
Clayton et al. [42]	2018	hiPSC-derived endothelial cells	Intradermal injection Suspended in medium and Matrigel	5 × 10^5^	Nude mice Non-diabetic	1. Increased angiogenesis 2. Accelerated wound closure 3. Increased collagen deposition, macrophage number, blood vessel density 4. Increased host expression of PECAM, Tie-1, and VEGF 5. Increased wound perfusion
Kim et al. [43]	2013	hiPSC-derived endothelial and smooth muscle cells	Intradermal injection in PBS	6 × 10^4^ EC + 4 × 10^4^ SMC	Nude mice Non-diabetic	1. Increased angiogenesis 2. Accelerated wound closure 3. Increased in vitro VEGF, EGF, and FGF-4 4. Increased smooth muscle cell migration
Shen et al. [48]	2016	hiPSC early vascular cells	Topical application Acrylated hyaluronic acid hydrogels	Variable	Nude mice STZ diabetic	1. Accelerated wound closure and perfusion 2. No significant difference between healthy and diabetic donor derived cells 3. Increased blood vessel density
Tan et al. [49]	2018	hiPSC-derived endothelial cells	Topical application Electrospun PCL/gelatin scaffolds	1 × 10^5^	FVB/N mice Non-diabetic	1. Increased angiogenesis compared to controls 2. Increased cell survival in scaffolds compared to cellular injections 3. Increased arteriole density in scaffold group compared to control and cellular injections
Kashpur et al. [55]	2018	hiPSC-derived fibroblasts	Topical application Polyethylene terephthalate membrane self-assembled tissues	16,000	Nude mice STZ diabetic	1. Accelerated wound healing with hiPSC-derived fibroblasts from DFU compared to primary cells 2. No difference in gene expression between hiPSC-derived fibroblasts derived from healthy and diabetic patients
Nakayama et al. [57]	2018	hiPSC-MSC	Intravenous injection	1 × 10^6^ and 3 × 10^5^	Nude mice Non-diabetic	1. Accelerated wound healing as measured by epithelialization after IV delivery of 1 × 10^6^ cells
Zhang et al. [59]	2015	hiPSC-MSC-derived extracellular vesicles	Intradermal injection + topical application in PBS	200 μg	SD rats Non-diabetic	1. Increased angiogenesis 2. Accelerated wound healing 3. Increased collagen density 4. Increased blood vessel density
Kobayashi et al. [60]	2018	hiPSC-derived extracellular vesicles	Intradermal injection + topical application in PBS	20 μg	C57 mice db/db diabetic	1. Increased angiogenesis 2. Accelerated wound healing 3. Increased in vitro fibroblasts migration and replication

Summary of studies implying induced pluripotent stem cells in cutaneous wound healing in a murine model, including cell type, delivery method, animal model, and major findings

*STZ* streptozocin, *SD* Sprague-Dawley

### Human-induced pluripotent stem cell-derived endothelial cells

Angiogenesis is a vital component of wound healing, as it reestablishes perfusion to injured tissues and delivers key nutrients. Unfortunately, angiogenesis is diminished in the hypoxic environment of chronic wounds. Additionally, diabetic patients have reduced homing potential of endothelial progenitor cells to sites of injury, further suppressing their angiogenic potential. Endothelial cells are critical for vessel formation and upregulation of VEGF expression. As such, delivery of human-induced pluripotent stem cell-derived endothelial cells (hiPSC-EC) holds great promise for accelerating diabetic wound healing [8, 9].

Several mechanisms by which hiPSC-EC improve wound healing have been identified thus far. Increases in wound perfusion and vessel density may be seen within the first 4 days following treatment in hiPSC-EC-treated wounds in a murine model [42]. hiPSC-EC-treated wounds have increased collagen deposition and macrophage number. Angiogenic gene expression, including endothelial cell adhesion molecule and VEGF, are also significantly upregulated. Cooperation of hiPSC-EC and human-induced pluripotent stem cell-derived smooth muscle cells (hiPSC-SMC) may also be important for neovascularization in dermal wounds. In vitro, hiPSC-EC elaborate more VEGF, epidermal growth factor (EGF), and FGF-4 compared to primary cells and are able to promote the chemotactic migration of smooth muscle cells [43]; in vivo, co-implantation of hiPSC-EC and hiPSC-SMC leads to greater vascular perfusion, significantly smaller open wound areas, and greater arteriole density compared with mice treated with hiPSC-EC alone [43].

The optimal delivery platform for iPSC and enhancement of their in vivo survival in chronic wounds is currently under investigation, with several biomimetic materials showing promise [48, 49]. The use of hyaluronic-acid (HA) hydrogel constructs containing hiPSC, endothelial progenitor, and early vascular cells has been shown to be an effective method of stem cell delivery [48]. These vascular constructs containing hiPSC derived from both healthy and type I diabetic patients accelerated the recruitment of host macrophages to the matrix and rapidly integrated into wound bed neovessels. Neovessels and macrophages in turn increased angiogenic factors, leading to increased angiogenesis and rapid wound closure.

One study showed there was no significant difference between healing in wounds treated with hiPSC derived from healthy versus type I diabetic donors in terms of both healing rate and time to reach maximum rate [48]. Although these findings need to be confirmed with cells from type II diabetics, these results hold promise for autologous transplant in diabetic patients. In murine models, iPSC-EC from obesity-induced diabetic mice showed defective function compared to iPSC-EC from healthy controls [50], suggesting further studies comparing iPSC derived from healthy and diabetic sources.

As with many stem cell approaches, the low rate of in vivo cell survival has been a major limitation in wound healing. The in vivo lifetime of hiPSC-EC increased by culturing them on electrospun polycaprolactone (PCL)/gelatin scaffolds; this mode of cell delivery also increased blood perfusion and arteriole density in the tissue surrounding hiPSC-EC-seeded scaffolds compared to controls [49]. Similar to observations by Shen et al. [48], the local immune response involving macrophages was increased by twofold in the presence of a scaffold alone, and this was further enhanced by the addition of hiPSC-EC, although macrophage subtype was not evaluated.

Together, these studies confirm that hiPSC-EC not only accelerate wound healing via increased angiogenesis, but have potential to do so to a greater extent than primary cells. Although equivocal in animal-derived iPSC, some studies examining hiPSC derived from diabetic humans suggest that they are not inferior to those from healthy donors, potentially allowing diabetic patients to undergo autologous transplant of their own cells with equal regenerative potential. This finding broadens the scope of hiPSC translational potential. Although the optimal delivery vehicle and hiPSC niche are yet to be elucidated, early studies show promising results at increasing cell survival in vivo, while advances in hiPSC-EC differentiation are becoming more efficient and rapid [51, 52].

### Human-induced pluripotent-derived fibroblasts

During wound healing, fibroblasts are responsible for the production of collagen, fibronectin, and proteoglycans, which form the extracellular matrix on which re-epithelialization and healing occur [53]. Fibroblasts isolated from DFU have decreased proliferation potential in response to growth factors [54], leading to delayed wound healing. As such, reconstitution of a healthy fibroblast population in DFU wounds can promote wound closure.

Kashpur et al. [55] found that hiPSC-derived fibroblasts from DFU are better at facilitating wound closure compared to primary DFU fibroblasts. hiPSC-derived cells from diabetic and healthy patients are more similar to each other than the cell lines from which they were derived and have similar gene expression. Gene ontology showed that hiPSC differ compared to primary cells in genes responsible for cell migration, cell proliferation, extracellular matrix organization, response to endogenous stimuli, developmental processes, and cell adhesion [55]. In functional assays, hiPSC-derived cells showed improved migratory properties in two-dimensional culture, although proliferation was unchanged [55].

In vivo, self-assembled 3D extracellular matrix tissues from hiPSC-derived fibroblasts improved re-epithelialization in a diabetic mouse model, when applied topically [55]. Tissues constructed from primary healthy foot fibroblasts significantly improved wound healing compared with primary fibroblasts of DFU origin. hiPSC derived from healthy and diabetic fibroblasts also accelerated wound healing to a greater extent than primary DFU-derived fibroblasts [55]. Therefore, hiPSC fibroblasts from diabetic subjects appear to have similar wound healing potential as those derived from healthy donors, suggesting their translational potential.

### Human-induced pluripotent-derived mesenchymal stem cells

MSC are multipotent stem cells that promote cutaneous healing by homing to wounds and differentiating into myogenic, chondrogenic, osteogenic, and adipogenic derivatives. MSC also produce growth hormones that drive angiogenesis and re-epithelialization, while mobilizing the resident stem cell niche and contributing to favorable immunomodulation [21]. However, MSC derived from diabetic rats appear to have impaired proliferation, differentiation, and expression of pro-angiogenic factors, and lack of ischemic wound healing potential [56]. Thus, repopulating chronic wounds with human-induced pluripotent stem cell-derived MSC (hiPSC-MSC) may have better potential to accelerate healing.

Nakayama et al. [57] successfully established hiPSC-MSC from keratinocytes of healthy individuals and patients with epidermolysis bullosa and studied their wound healing potential in a nude mouse model [57]. With subcutaneous and intravenous delivery, hiPSC-MSC successfully secreted human type VII collagen at the dermal-epidermal junction. Intravenous injection of cells led to increased wound healing as measured by epithelialization. However, cells were eliminated from the wounds by 2 weeks.

### Human-induced pluripotent stem cell-derived extracellular vesicles

Although pluripotent iPSC hold tremendous promise in wound healing and regenerative medicine, their use in clinical trials is currently limited due to their teratogenic potential. Although teratoma formation after topical transplantation has not yet been studied, subcutaneous injection of differentiated iPSC led to teratoma formation [58]. Thus, harnessing hiPSC potential independent of cellular transfer may prove to be safer. Nano-sized extracellular vesicles containing protein, mRNA, and miRNA (previously known as exosomes) accelerate chronic wound healing similar to hiPSC, but eliminate the risks of teratoma formation as they are devoid of nuclei and therefore incapable of dividing, making them a new and exciting candidate among potential wound healing therapies.

Extracellular vesicles released from hiPSC-MSC facilitate cutaneous wound healing by promoting collagen synthesis and angiogenesis [59, 60]. Specifically, hiPSC-MSC extracellular vesicle treatment promotes greater wound closure, faster re-epithelialization, decreased scar width, and higher density of blood vessels in treated animals including diabetic wound models [59, 60]. In vitro, human fibroblasts show greater proliferation, migration, and expression of fibronectin, collagen type I and III, and elastin in the presence of hiPSC-MSC-extracellular vesicles. Similarly, human umbilical vein endothelial cell (HUVEC) exhibit increased migration, proliferation, tubule formation, and branching with hiPSC-MSC-extracellular vesicle treatment [59].

The primary hurdle to overcome before clinical application of hiPSC-derived cells is the elimination of their tumorigenic potential. The aforementioned studies were able to harness the therapeutic potential of hiPSC without obvious negative side effects, a major step in furthering these cells’ translational potential.

## Human-induced pluripotent stem cells in ischemic wounds

In addition to their therapeutic potential in cutaneous wound healing, iPSC are currently being investigated in multiple disease models of ischemia, including peripheral arterial disease, myocardial infarction, osteonecrosis, and retinopathy. Consistent with findings from cutaneous wound healing studies, the primary driver of iPSC healing in the majority of these models is their pro-angiogenic potential [61]. Although iPSC have thus far been examined in models of peripheral arterial disease, these models have not yet evaluated wound healing in chronically ischemic limbs.

iPSC ameliorate ischemia in models of peripheral arterial disease by reducing inflammation, promoting angiogenesis, and reconstituting viable cellular populations such as endothelial cells. In a nude mouse hind limb ischemia model, hiPSC-EC in conjunction with VEGF reduce inflammation and promote muscular regeneration [62]. hiPSC-EC also improved blood flow to ischemic limbs by increasing the total number of capillaries as well as angiogenic cytokines and growth factors [63]. Compared to induced endothelial cells generated from fibroblasts, hiPSC-EC show similar perfusion recovery, although capillary density in the ischemic muscle was only increased in the induced endothelial cell group [64]. Furthermore, hiPSC-EC enhanced angiogenesis via paracrine signaling in ischemic tissue [65].

In a similar model, when compared to bone marrow-derived MSC, hiPSC-MSC reduced muscle fibrosis, improved ambulatory impairment and tissue loss, and enhanced perfusion [66]. hiPSC-MSC extracellular vesicles also enhance microvessel density and blood perfusion in limb ischemia models. In vitro, these extracellular vesicles activated angiogenesis-related protein expression and promoted HUVEC migration, proliferation, and tubule formation [67]. Similarly, hiPSC-derived pericytes injected into ischemic murine limbs increased the reperfusion of limbs by approximately fourfold by incorporating not only into host vasculature, but also into muscle [68]. This observation demonstrates their potential for differentiation and regeneration of multiple types of ischemic tissue.

The majority of studies employing iPSC in models of peripheral arterial disease have delivered cells or extracellular vesicles via intramuscular (IM) injection with cells suspended in buffer or culture medium [61, 63–69]. Cell survival was increased when the cells were suspended in a recombinant hydrogel, which may prevent cell membrane damage from the shear stress of injection [70]. Similarly, delivery of cells in a shear-thinning hydrogel for injectable encapsulation and long-term delivery (SHIELD) found that cells were not only protected from syringe shear stress resulting in higher acute cell survival, but had enhanced in vivo retention [70]. Similar to studies of iPSC in cutaneous wound healing, the optimal delivery method for cells in models of peripheral arterial disease remains to be perfected.

## Induced pluripotent stem cells and teratoma formation

Despite their great promise, the use of pluripotent stem cells whether induced or embryonic is limited by their tumorigenic potential [71–73]. Because iPSC and human embryonic cells are capable of differentiating into cells from any of the three germ layers, they also carry the potential to form teratomas in the undifferentiated state. For example, in vivo teratoma formation in immunodeficient mice is frequently used as an assay for defining pluripotency [74–76]. Because hiPSC and their behavior are complex, the latency, efficiency, and tissue composition of their resultant tumors vary greatly with the number of transplanted cells, site of injection, cell line, and mode of hiPSC derivation. Even among ten commercially available hiPSC cell lines derived in a similar fashion, there were variations in tumor incidence, formation latency, and tumor volumes; these differences may be attributed to different viral insertion sites, as well as acquired mutations [69].

Numerous strategies are currently under investigation aimed at eliminating iPSC teratogenic potential, in addition to the use of extracellular vesicles mentioned above. Cellular differentiation prior to cell transplantation is one approach to decreasing the risk of teratoma formation. For example, cellular-based therapies, such as those used in some wound healing studies, use terminally differentiated cells. This strategy, however, does not eliminate the risk of inadvertent transplantation of residual undifferentiated cells. In fact, multiple studies using neural and chondrogenic derivatives have shown persistent teratoma formation [58, 77, 78]. Further, gene expression patterns from hiPSC-MSC overlap with those from cancer cells, but not corresponding primary cells [79].

Several methods are currently employed for generating iPSC, with the oncogenic safety profile of some superior to others (Table 1). Retroviral vectors were the first to be described, including Moloney murine leukemia virus (MMLV) and lentivirus. However, these viruses integrate into the host genome and require the use of harmful viral particles expressing oncogenes. They are also prone to insertional mutagenesis and are therefore not safe for clinical application. The piggyback transposon, although still integrative, can be excised. Adenovirus, Sendai virus, and plasmid delivered vectors do not integrate into the host genome, but are less efficient and difficult to clear from host cells. Plasmids, small plasmids, and episomes are safer than viral vectors, but some genome integration has been observed. By far, the safest reprogramming methods are those which employ the use of RNA, protein, and small particle chemical delivery, all of which are transgene-free. Unfortunately, these methods are the least efficient and slowest; nonetheless, they are being employed with greater frequency [34, 35].

Other modes of eliminating iPSC oncogenic potential are being investigated. Cells are now successfully reprogrammed without the use of c-Myc, the most oncogenic Yamanaka factor [80]. Small molecules including quercetin and YM155, which target anti-apoptotic signals such as survivin, have been successfully used to eliminate the undifferentiated cells in a mixed population in vitro [81]. Similarly, inhibitors of lysine-specific demethylase 1, deregulated in teratogenic cells, prevent tumor formation [82]. Pluripotent cell-specific inhibitors (PluriSIns), including oleic acid synthesis inhibitors, are now commercially available for the prevention of teratoma formation after the use of undifferentiated cells [83]. Undifferentiated cells capable of teratoma formation can also be distinguished morphologically from their differentiated counterparts to select for differentiated cells only [84]. Brentuximab vedotin, which targets CD30 on undifferentiated cells and induces apoptosis, has been used to eliminate the teratogenic potential of iPSC-derived cardiomyocytes [85]. Together, these techniques have the potential to produce iPSC in safer and more efficient ways, hastening their entry into clinical trials.

## Conclusion

iPSC are an innovative and exciting new cell type with potential to revolutionize the fields of regenerative medicine, inherited genetic disease, and drug therapy. hiPSC hold great promise to accelerate chronic wound healing, with increased healing and reperfusion following wounding or ischemia in rodent preclinical models. Although much has been learned about iPSC generation and optimization in the short time since their development, their safety profile, particularly in relation to tumorigenic potential, remains to be understood in sufficient detail to allow clinical translation. In addition, hiPSC can be derived from patients with chronic diseases and reprogrammed into cells functionally resembling those derived from healthy individuals. However, iPSC-EC derived from obesity-induced diabetic mice showed decreased healing and angiogenic capacity, and thus, further studies will be required to understand the differences between hiPSC derived from diseased and healthy donors.

Major hurdles must be overcome before iPSC use in humans becomes possible. Methods for generating hiPSC with non-integrative technology, preferably by protein and small particle transfer, must become more efficient and speedier. Standardized protocols for teratoma formation assays as proof of pluripotency must be developed in order to derive meaningful conclusions about cell lines. The optimal platform for delivery of hiPSC into a wound, including the ideal niche to prolong cell survival and intracellular signaling, needs to be determined. New methods for eliminating undifferentiated cells capable of tumorigenic potential prior to cellular transplant are necessary. Although studies have examined the tumorigenic potential of cells injected directly into murine tissues, no study to date has examined this potential after cutaneous application of hiPSC. Thus, further animal studies to evaluate the safety and efficacy of iPSC are crucial prior to their translation into humans, including large animal models that more closely mimic human skin [86]. Lastly, since the therapeutic potential of hiPSC appears to be mainly driven by their paracrine effects, the role of extracellular vesicles, which entirely eliminate tumorigenic potential, should be understood.

## Abbreviations

3D: Three-dimensional
DFU: Diabetic foot ulcer
EGF: Epidermal growth factor
ESC: Embryonic stem cell
FGF: Fibroblast growth factor
HA: Hyaluronic acid
hiPSC-EC: Human-induced pluripotent stem cell-derived endothelial cell
hiPSC-MSC: Human-induced pluripotent stem cell-derived mesenchymal stem cell
hiPSC-SMC: Human-induced pluripotent stem cell-derived smooth muscle cell
HUVEC: Human umbilical vein endothelial cell
IL-1: Interleukin 1
IM: Intramuscular
iPSC: Induced pluripotent stem cell
MMLV: Moloney murine leukemia virus
MSC: Mesenchymal stem cell
PCL: Polycaprolactone
PDGF: Platelet-derived growth factor
SHIELD: Shear-thinning hydrogel for injectable encapsulation and long-term delivery
TNF-α: Tumor necrosis factor alpha
VEGF: Vascular endothelial growth factor

## References

[1] Falanga V (2005). Wound healing and its impairment in the diabetic foot. Lancet.

[2] American Diabetes Association (2014). National Diabetes Statistics Report.

[3] Eming SA, Martin P, Tomic-Canic M (2014). Wound repair and regeneration: mechanisms, signaling, and translation. Sci Transl Med.

[4] Martin P, Nunan R (2015). Cellular and molecular mechanisms of repair in acute and chronic wound healing. Br J Dermatol.

[5] Werner S, Grose R (2003). Regulation of wound healing by growth factors and cytokines. Physiol Rev.

[6] American Diabetes A (2013). Economic costs of diabetes in the U.S. in 2012. Diabetes Care.

[7] Fox JD, Baquerizo Nole KL, Berriman SJ, Kirsner RS (2016). Chronic wounds: the need for greater emphasis in medical schools, post-graduate training and public health discussions. Ann Surg.

[8] Gallagher KA, Liu Z-J, Xiao M, Chen H, Goldstein LJ, Buerk DG (2007). Diabetic impairments in NO-mediated endothelial progenitor cell mobilization and homing are reversed by hyperoxia and SDF-1 alpha. J Clin Invest.

[9] Galkowska H, Wojewodzka U, Olszewski WL (2006). Chemokines, cytokines, and growth factors in keratinocytes and dermal endothelial cells in the margin of chronic diabetic foot ulcers. Wound Repair Regen.

[10] Galiano RD, Tepper OM, Pelo CR, Bhatt KA, Callaghan M, Bastidas N (2004). Topical vascular endothelial growth factor accelerates diabetic wound healing through increased angiogenesis and by mobilizing and recruiting bone marrow-derived cells. Am J Pathol.

[11] Maruyama K, Asai J, Ii M, Thorne T, Losordo DW, D'Amore PA (2007). Decreased macrophage number and activation lead to reduced lymphatic vessel formation and contribute to impaired diabetic wound healing. Am J Pathol.

[12] Tecilazich F, Dinh T, Veves A (2011). Treating diabetic ulcers. Expert Opin Pharmacother.

[13] Cavanagh PR, Lipsky BA, Bradbury AW, Botek G (2005). Treatment for diabetic foot ulcers. Lancet.

[14] Frykberg RG, Zgonis T, Armstrong DG, Driver VR, Giurini JM, Kravitz SR (2006). Diabetic foot disorders: a clinical practice guideline (2006 revision). J Foot Ankle Surg.

[15] Holmes C, Wrobel JS, Maceachern MP, Boles BR (2013). Collagen-based wound dressings for the treatment of diabetes-related foot ulcers: a systematic review. Diabetes Metab Syndr Obes.

[16] Sheehan P, Jones P, Caselli A, Giurini JM, Veves A (2003). Percent change in wound area of diabetic foot ulcers over a 4-week period is a robust predictor of complete healing in a 12-week prospective trial. Diabetes Care.

[17] Isakson M, de Blacam C, Whelan D, McArdle A, Clover AJP (2015). Mesenchymal stem cells and cutaneous wound healing: current evidence and future potential. Stem Cells Int.

[18] Cao Y, Gang X, Sun C, Wang G (2017). Mesenchymal stem cells improve healing of diabetic foot ulcer. J Diabetes Res.

[19] Mizukami H, Yagihashi S (2014). Exploring a new therapy for diabetic polyneuropathy - the application of stem cell transplantation. Front Endocrinol.

[20] Jiang X-Y, Lu D-B, Chen B (2012). Progress in stem cell therapy for the diabetic foot. Diabetes Res Clin Pract.

[21] Balaji S, Keswani SG, Crombleholme TM (2012). The role of mesenchymal stem cells in the regenerative wound healing phenotype. Adv Wound Care.

[22] Blumberg SN, Berger A, Hwang L, Pastar I, Warren SM, Chen W (2012). The role of stem cells in the treatment of diabetic foot ulcers. Diabetes Res Clin Pract.

[23] Wang S, Qu X, Zhao RC (2012). Clinical applications of mesenchymal stem cells. J Hematol Oncol.

[24] Sorrell JM, Caplan AI (2010). Topical delivery of mesenchymal stem cells and their function in wounds. Stem Cell Res Ther.

[25] Lu D, Chen B, Liang Z, Deng W, Jiang Y, Li S (2011). Comparison of bone marrow mesenchymal stem cells with bone marrow-derived mononuclear cells for treatment of diabetic critical limb ischemia and foot ulcer: a double-blind, randomized, controlled trial. Diabetes Res Clin Pract.

[26] Xu S-M, Liang T (2016). Clinical observation of the application of autologous peripheral blood stem cell transplantation for the treatment of diabetic foot gangrene. Exp Ther Med.

[27] Kirana S, Stratmann B, Prante C, Prohaska W, Koerperich H, Lammers D (2012). Autologous stem cell therapy in the treatment of limb ischaemia induced chronic tissue ulcers of diabetic foot patients. Int J Clin Pract.

[28] Kimbrel EA, Kouris NA, Yavanian GJ, Chu J, Qin Y, Chan A (2014). Mesenchymal stem cell population derived from human pluripotent stem cells displays potent immunomodulatory and therapeutic properties. Stem Cells Dev.

[29] Wu DC, Boyd AS, Wood KJ (2007). Embryonic stem cell transplantation: potential applicability in cell replacement therapy and regenerative medicine. Front Biosci.

[30] Takahashi K, Tanabe K, Ohnuki M, Narita M, Ichisaka T, Tomoda K (2007). Induction of pluripotent stem cells from adult human fibroblasts by defined factors. Cell..

[31] Takahashi K, Yamanaka S (2006). Induction of pluripotent stem cells from mouse embryonic and adult fibroblast cultures by defined factors. Cell..

[32] Yu J, Vodyanik MA, Smuga-Otto K, Antosiewicz-Bourget J, Frane JL, Tian S (2007). Induced pluripotent stem cell lines derived from human somatic cells. Science..

[33] Haridhasapavalan KK, Borgohain MP, Dey C, Saha B, Narayan G, Kumar S (2019). An insight into non-integrative gene delivery approaches to generate transgene-free induced pluripotent stem cells. Gene..

[34] Malik N, Rao MS (2013). A review of the methods for human iPSC derivation. Methods Mol Biol.

[35] Deng X-Y, Wang H, Wang T, Fang X-T, Zou L-L, Li Z-Y (2015). Non-viral methods for generating integration-free, induced pluripotent stem cells. Curr Stem Cell Res Ther.

[36] Ojeh N, Pastar I, Tomic-Canic M, Stojadinovic O (2015). Stem cells in skin regeneration, wound healing, and their clinical applications. Int J Mol Sci.

[37] Kirby GT, Mills SJ, Cowin AJ, Smith LE (2015). Stem cells for cutaneous wound healing. Biomed Res Int.

[38] Singh VK, Kalsan M, Kumar N, Saini A, Chandra R (2015). Induced pluripotent stem cells: applications in regenerative medicine, disease modeling, and drug discovery. Front Cell Dev Biol.

[39] Baraniak PR, McDevitt TC (2010). Stem cell paracrine actions and tissue regeneration. Regen Med.

[40] Liang X, Ding Y, Zhang Y, Tse H-F, Lian Q (2014). Paracrine mechanisms of mesenchymal stem cell-based therapy: current status and perspectives. Cell Transplant.

[41] Casqueiro J, Casqueiro J, Alves C (2012). Infections in patients with diabetes mellitus: a review of pathogenesis. Indian J Endocrinol Metab.

[42] Clayton ZE, Tan RP, Miravet MM, Lennartsson K, Cooke JP, Bursill CA (2018). Induced pluripotent stem cell-derived endothelial cells promote angiogenesis and accelerate wound closure in a murine excisional wound healing model. Biosci Rep.

[43] Kim KL, Song SH, Choi KS, Suh W (2013). Cooperation of endothelial and smooth muscle cells derived from human induced pluripotent stem cells enhances neovascularization in dermal wounds. Tissue Eng Part A.

[44] Açikgoz G, Devrim İ, Özdamar Ş (2004). Comparison of keratinocyte proliferation in diabetic and non-diabetic inflamed gingiva. J Periodontol.

[45] Liu X, Li Q, Niu X, Hu B, Chen S, Song W (2017). Exosomes secreted from human-induced pluripotent stem cell-derived mesenchymal stem cells prevent osteonecrosis of the femoral head by promoting angiogenesis. Int J Biol Sci.

[46] Itoh M, Umegaki-Arao N, Guo Z, Liu L, Higgins CA, Christiano AM (2013). Generation of 3D skin equivalents fully reconstituted from human induced pluripotent stem cells (iPSCs). PLoS One.

[47] Kuzuya M, Satake S, Esaki T, Yamada K, Hayashi T, Naito M (1995). Induction of angiogenesis by smooth muscle cell-derived factor: possible role in neovascularization in atherosclerotic plaque. J Cell Physiol.

[48] Shen YI, Cho H, Papa AE, Burke JA, Chan XY, Duh EJ (2016). Engineered human vascularized constructs accelerate diabetic wound healing. Biomaterials..

[49] Tan RP, Chan AHP, Lennartsson K, Miravet MM, Lee BSL, Rnjak-Kovacina J (2018). Integration of induced pluripotent stem cell-derived endothelial cells with polycaprolactone/gelatin-based electrospun scaffolds for enhanced therapeutic angiogenesis. Stem Cell Res Ther.

[50] Gu M, Mordwinkin NM, Kooreman NG, Lee J, Wu H, Hu S (2015). Pravastatin reverses obesity-induced dysfunction of induced pluripotent stem cell-derived endothelial cells via a nitric oxide-dependent mechanism. Eur Heart J.

[51] Patsch C, Challet-Meylan L, Thoma EC, Urich E, Heckel T, O'Sullivan JF (2015). Generation of vascular endothelial and smooth muscle cells from human pluripotent stem cells. Nat Cell Biol.

[52] Prasain N, Lee MR, Vemula S, Meador JL, Yoshimoto M, Ferkowicz MJ (2014). Differentiation of human pluripotent stem cells to cells similar to cord-blood endothelial colony-forming cells. Nat Biotechnol.

[53] Dash BC, Xu Z, Lin L, Koo A, Ndon S, Berthiaume F (2018). Stem cells and engineered scaffolds for regenerative wound healing. Bioengineering (Basel).

[54] Loots MAM, Kenter SB, Au FL, van Galen WJM, Middelkoop E, Bos JD (2002). Fibroblasts derived from chronic diabetic ulcers differ in their response to stimulation with EGF, IGF-I, bFGF and PDGF-AB compared to controls. Eur J Cell Biol.

[55] Kashpur O, Smith A, Gerami-Naini B, Maione AG, Calabrese R, Tellechea A, et al. Differentiation of diabetic foot ulcer-derived induced pluripotent stem cells reveals distinct cellular and tissue phenotypes. FASEB J. 2018. 10.1096/fj.201801059. 10.1096/fj.201801059 PMC6355091 30088952

[56] Kim H, Han JW, Lee JY, Choi YJ, Sohn Y-D, Song M (2015). Diabetic mesenchymal stem cells are ineffective for improving limb ischemia due to their impaired angiogenic capability. Cell Transplant.

[57] Nakayama C, Fujita Y, Matsumura W, Ujiie I, Takashima S, Shinkuma S (2018). The development of induced pluripotent stem cell-derived mesenchymal stem/stromal cells from normal human and RDEB epidermal keratinocytes. J Dermatol Sci.

[58] Roy NS, Cleren C, Singh SK, Yang L, Beal MF, Goldman SA (2006). Functional engraftment of human ES cell–derived dopaminergic neurons enriched by coculture with telomerase-immortalized midbrain astrocytes. Nat Med.

[59] Zhang J, Guan J, Niu X, Hu G, Guo S, Li Q (2015). Exosomes released from human induced pluripotent stem cells-derived MSCs facilitate cutaneous wound healing by promoting collagen synthesis and angiogenesis. J Transl Med.

[60] Kobayashi H, Ebisawa K, Kambe M, Kasai T, Suga H, Nakamura K, et al. Editors’ Choice Effects of exosomes derived from the induced pluripotent stem cells on skin wound healing. Nagoya J Med Sci 2018;80(2):141–153. 10.18999/nagjms.80.2.141 PMC5995743 29915432

[61] Lai W-H, Ho JCY, Chan Y-C, Ng JHL, Au K-W, Wong L-Y (2013). Attenuation of hind-limb ischemia in mice with endothelial-like cells derived from different sources of human stem cells. PloS One.

[62] Mulyasasmita W, Cai L, Dewi RE, Jha A, Ullmann SD, Luong RH (2014). Avidity-controlled hydrogels for injectable co-delivery of induced pluripotent stem cell-derived endothelial cells and growth factors. J Control Release.

[63] Rufaihah AJ, Huang NF, Jame S, Lee JC, Nguyen HN, Byers B (2011). Endothelial cells derived from human iPSCS increase capillary density and improve perfusion in a mouse model of peripheral arterial disease. Arterioscler Thromb Vasc Biol.

[64] Clayton ZE, Yuen GS, Sadeghipour S, Hywood JD, Wong JW, Huang NF (2017). A comparison of the pro-angiogenic potential of human induced pluripotent stem cell derived endothelial cells and induced endothelial cells in a murine model of peripheral arterial disease. Int J Cardiol.

[65] Yoo CH, Na H-J, Lee D-S, Heo SC, An Y, Cha J (2013). Endothelial progenitor cells from human dental pulp-derived iPS cells as a therapeutic target for ischemic vascular diseases. Biomaterials..

[66] Lian Q, Zhang Y, Zhang J, Zhang HK, Wu X, Zhang Y (2010). Functional mesenchymal stem cells derived from human induced pluripotent stem cells attenuate limb ischemia in mice. Circulation..

[67] Hu GW, Li Q, Niu X, Hu B, Liu J, Zhou SM (2015). Exosomes secreted by human-induced pluripotent stem cell-derived mesenchymal stem cells attenuate limb ischemia by promoting angiogenesis in mice. Stem Cell Res Ther.

[68] Dar A, Domev H, Ben-Yosef O, Tzukerman M, Zeevi-Levin N, Novak A (2012). Multipotent vasculogenic pericytes from human pluripotent stem cells promote recovery of murine ischemic limb. Circulation..

[69] Yasuda S, Kusakawa S, Kuroda T, Miura T, Tano K, Takada N (2018). Tumorigenicity-associated characteristics of human iPS cell lines. PLoS One.

[70] Foster AA (2018). Protein-engineered hydrogels enhance the survival of induced pluripotent stem cell-derived endothelial cells for treatment of peripheral arterial disease. Biomater Sci.

[71] Gutierrez-Aranda I, Ramos-Mejia V, Bueno C, Munoz-Lopez M, Real PJ, Macia A (2010). Human induced pluripotent stem cells develop teratoma more efficiently and faster than human embryonic stem cells regardless the site of injection. Stem Cells.

[72] Zhang Y, Wang D, Chen M, Yang B, Zhang F, Cao K (2011). Intramyocardial transplantation of undifferentiated rat induced pluripotent stem cells causes tumorigenesis in the heart. PLoS One.

[73] Gerami-Naini B, Smith A, Maione AG, Kashpur O, Carpinito G, Veves A (2016). Generation of induced pluripotent stem cells from diabetic foot ulcer fibroblasts using a nonintegrative Sendai virus. Cell Reprogram.

[74] Przyborski SA (2005). Differentiation of human embryonic stem cells after transplantation in immune-deficient mice. Stem Cells.

[75] Prokhorova TA, Harkness LM, Frandsen U, Ditzel N, Schrøder HD, Burns JS (2009). Teratoma formation by human embryonic stem cells is site dependent and enhanced by the presence of Matrigel. Stem Cells Dev.

[76] Cooke MJ, Stojkovic M, Przyborski DSA (2006). Growth of teratomas derived from human pluripotent stem cells is influenced by the graft site. Stem Cells Dev.

[77] Wernig M, Benninger F, Schmandt T, Rade M, Tucker KL, Büssow H (2004). Functional integration of embryonic stem cell-derived neurons <em>in vivo</em&gt. J Neurosci.

[78] Saito T, Yano F, Mori D, Kawata M, Hoshi K, Takato T (2015). Hyaline cartilage formation and tumorigenesis of implanted tissues derived from human induced pluripotent stem cells. Biomed Res.

[79] Zhao Q, Gregory CA, Lee RH, Reger RL, Qin L, Hai B (2015). MSCs derived from iPSCs with a modified protocol are tumor-tropic but have much less potential to promote tumors than bone marrow MSCs. Proc Natl Acad Sci U S A.

[80] Wernig M, Meissner A, Cassady JP, Jaenisch R (2008). C-Myc is dispensable for direct reprogramming of mouse fibroblasts. Cell Stem Cell.

[81] Lee MO, Moon SH, Jeong HC, Yi JY, Lee TH, Shim SH (2013). Inhibition of pluripotent stem cell-derived teratoma formation by small molecules. Proc Natl Acad Sci U S A.

[82] Osada N, Kikuchi J, Umehara T, Sato S, Urabe M, Abe T (2018). Lysine-specific demethylase 1 inhibitors prevent teratoma development from human induced pluripotent stem cells. Oncotarget..

[83] Ben-David U, Gan Q-F, Golan-Lev T, Arora P, Yanuka O, Oren Yifat S (2013). Selective elimination of human pluripotent stem cells by an oleate synthesis inhibitor discovered in a high-throughput screen. Cell Stem Cell.

[84] Nishimori M, Yakushiji H, Mori M, Miyamoto T, Yaguchi T, Ohno S (2014). Tumorigenesis in cells derived from induced pluripotent stem cells. Hum Cell.

[85] Sougawa N, Miyagawa S, Fukushima S, Kawamura A, Yokoyama J, Ito E (2018). Immunologic targeting of CD30 eliminates tumourigenic human pluripotent stem cells, allowing safer clinical application of hiPSC-based cell therapy. Sci Rep.

[86] Grada A, Mervis J, Falanga V (2018). Research techniques made simple: animal models of wound healing. J Invest Dermatol.

